# Revealing the Impact of Viscoelastic Characteristics on Performance Parameters of UV-Crosslinked Hotmelt Pressure-Sensitive Adhesives: Insights from Time–Temperature Superposition Analysis

**DOI:** 10.3390/polym16152123

**Published:** 2024-07-25

**Authors:** Marian Guder, Roman Günther, Katharina Bremgartner, Nicole Senn, Christof Brändli

**Affiliations:** 1 Laboratory of Adhesives and Polymer Materials, Institute of Materials and Process Engineering, ZHAW Zurich University of Applied Sciences, 8401 Winterthur, Switzerland; marian.guder@aquatherm.de (M.G.); roman.guenther@protonmail.com (R.G.); 2 aquatherm GmbH, 57439 Attendorn, Germany; 3 Artimelt AG, 6210 Sursee, Switzerlandnicole.senn@artimelt.com (N.S.)

**Keywords:** pressure-sensitive adhesives (PSAs), viscoelastic properties, UV-triggered crosslinking, time–temperature superposition (TTS), rheology

## Abstract

This study emphasizes the influential role of rheology in decoding the viscoelastic properties of pressure-sensitive adhesives (PSAs) vital to predicting key application features such as shear, tack, and peel, depending on the flow characteristics of PSAs during bonding and debonding processes. By applying the principle of time–temperature superposition (TTS), we extend the scope of our frequency analysis, surpassing the technical constraints of the available apparatus. Our exploration aims to uncover the general correlations between PSAs’ viscoelastic properties and their performance in end-use applications. Initially, the adhesive performance and viscoelastic properties of a UV-crosslinkable styrene-butadiene-styrene (SBS) model adhesive prior and subsequent to UV irradiation were examined. The subsequent crosslinking reaction increased cohesive strength and heat resistance, although tack and peel strength observed a substantial decline. We successfully demonstrated these effects by logging the viscoelastic properties, specifically the storage modulus G′ at lower frequencies, which mirrors the shear strength at higher temperatures and the shift in the tan δ peak to represent each PSA’s tack. These correlations were partially reflected in three commercial UV crosslinkable acrylic PSA products, although the effect of UV irradiation was less distinctive. This study also revealed the challenges in predicting tack and peel strength, which result from a complex interplay of bonding and debonding processes. Our findings reinforce the necessity for more sophisticated analysis techniques and models that can accurately predict the end-use performance of PSAs across different physical structures and chemical compositions. Further research is needed to develop these predictive models, which may reduce the need for labor-intensive testing under real-life conditions.

## 1. Introduction

Pressure-sensitive adhesives (PSAs) penetrate our daily life with their usefulness for a multitude of applications since their introduction over a century ago. PSAs possess distinctive properties that differentiate them from other adhesive types, rendering them suitable for diverse applications, ranging from office supplies in the packaging sector to medical products. Contrary to other adhesives, PSAs do not necessarily undergo chemical or physical alterations during the bonding process. This unique quality enables PSAs to exhibit both solid-like and fluid-like behavior pre- and post-bond formation [1,2,3].

Despite the inherent cohesiveness of PSAs, which eliminates the need for chemical reactions to form strong bonds, recent advances introduced by BASF researchers have incorporated the concept of UV crosslinking in PSAs. This technique involves radiation-induced crosslinking of these adhesives, which enables tunable end-use properties by modulating the UV energy dose post-coating. A higher UV exposure results in a greater crosslink density and thus enhanced cohesive strength, albeit at the expense of tack and adhesion [4,5,6]. Furthermore, UV exposure impacts the viscoelastic behavior of materials, affecting both storage modulus and loss modulus. It is anticipated that both moduli will increase, with the storage modulus eventually surpassing the loss modulus.

Considering the labor-intensive and time-consuming nature of evaluating application properties, we pursued the identification of analytical methods to accurately forecast the final properties of UV-crosslinkable PSAs. Dynamic mechanical analysis (DMA) via rheological evaluation emerged as the most promising strategy due to several advantages. Firstly, the flow behavior of PSAs significantly correlates with their end-use performance, since they exist in a viscoelastic state under application conditions [7,8]. Secondly, the methodology of rheology offers the following practical benefits: it assesses bulk materials, eliminating the need to prepare test specimens by coating the adhesive to a carrier, thereby allowing for high-throughput testing multiple formulations and curing conditions.

The time–temperature superposition (TTS) principle suggests that the influence of time and temperature on viscoelastic materials can be superposed. The core idea revolves around the understanding that the ratio between the recoverable energy during a given deformation and the energy losses in the same process depends (a) on the duration the stress is applied (stress rate, frequency) and (b) on the temperature, as an increase facilitates molecular relaxation and rearrangements [1,9]. Using the TTS principle, by varying the temperature, we can draw conclusions about the influence of time or—in the case of DMA—about the influence of frequency as the reciprocal of time.

There have been numerous instances of employing the TTS principle in the DMA of PSAs [7,10,11,12,13,14,15]. The application of DMA [16,17,18,19] and TTS [20,21] has also extended to the realm of UV-crosslinkable materials. However, the consistency of some results has been under scrutiny, and the applicability of the findings from customized and laboratory-produced adhesives of similar composition to complex industrial products is yet to be conclusively demonstrated. In addition, the findings to date are often limited to just one material class. It must therefore be examined to what extent the results of adhesives of different basic chemistry can be compared and summarized in a common model.

Therefore, the objectives of this study are three-fold: (1) to develop a robust and reproducible method for determining the viscoelastic properties of UV-crosslinkable PSA materials; (2) to establish correlations between viscoelastic properties and the final performance of the materials, specifically shear, peel, and tack performance; and (3) to affirm the applicability of these results to commercially available, real-world PSA products of different base polymers. For this purpose, we investigated one styrene–butadiene–styrene (SBS) model adhesive and three commercial acrylic adhesives, each differing in their application properties. Additionally, we evaluated the influence of UV-initiated crosslinking based on the model adhesive.

## 2. Materials and Methods

### 2.1. Materials

One styrene–butadiene–styrene (SBS) block copolymer model adhesive formulation and three commercially available UV-crosslinkable acrylic hotmelt PSAs differing in their adhesive properties (high-tack, high-shear, intermediate) were kindly provided by artimelt AG, Sursee, Switzerland. The model SBS adhesive was mixed at 140 °C with 2 parts per hundred of rubber (phr) photoinitiator Genocure TPO-L (Rahn AG, Zürich, Switzerland) and mixed for a total of 330 s at 2500 rounds per minute (rpm) using a Hauschild SpeedMixer DAC400 (Hamm, Germany). The resulting formulation was used as a reference material for the development of an appropriate rheological methodology for DMA. The three commercial acrylic adhesives did already contain a suitable photoinitiator, and were used as received.

### 2.2. Rheological Evaluation

The Anton Paar MCR 302 Rheometer (Graz, Austria) with a parallel plate geometry of 25 mm in diameter (PP25) was used for rheological assessments. The lower plate was fitted with quartz glass, enabling direct irradiation via the Omnicure Series S2000 Spot UV Curing System (Excelitas Technologies Corp., Waltham, MA, USA). Frequency sweeps were conducted at a constant strain of 0.1% and a normal axial force of 1 N between 0.1 and 100 Hz at temperatures from −10 (above Tg) to 150 °C. All materials were tested before and after UV irradiation for 600 s, which resulted in a total dose of 5000 mJ/cm^2^ between 200 and 400 nm.

Time–temperature superposition evaluations were performed using the TRIOS software 5.0 from TA Instruments (Eschborn, Germany), applying the Williams–Landel–Ferry (WLF) model.

### 2.3. Specimen Preparation

PSA materials were coated onto a 50 µm thick PET film at a weight of 25 g/m^2^ using the Acumeter LH-3 laboratory coating laminator (Acumeter Laboratories Inc., St. Paul, MN, USA). Test specimens were then cut from this film for shear, tack, and peel tests. When UV treatment was required, the coated samples were exposed to irradiation on an Uviterno BT-3015 (Uviterno AG, Berneck, Switzerland) belt dryer with a total dose of 5000 mJ/cm^2^ between 200 and 400 nm.

### 2.4. Shear Adhesion Failure Temperature (SAFT)

Shear adhesion failure temperature (SAFT) was tested as per PSTC-17. Test specimens, each measuring 25 mm in width and 100 mm in length (in the machine direction), were cut from the coated PET layer. One end of each specimen was centered on a stainless steel plate, covering an area of 25 × 25 mm^2^. A specified pressure was applied by rolling down the test area twice in each lengthwise direction using a 2 kg roller at a rolling speed of 10 mm/s. After a wet-out period of a minimum of four hours under ambient conditions, the samples were mounted vertically in a programmable convection oven, and a 1000 g load was applied. Starting at 20 °C, the oven temperature was increased by 0.5 °C per minute. The time until the specimen separated from the steel plate was recorded and used for the SAFT calculation.

### 2.5. Peel Test

Peel tests were conducted on a Zwick Roell zwickiLine Z5.0 TN universal testing machine (ZwickRoell GmbH & Co. KG, Ulm, Germany) in accordance with PSTC-101. Test specimens, each 25 mm wide and 300 mm long (in machine direction), were prepared from the coated PET layer. One end of each specimen was positioned on a stainless steel plate covering an area of 25 × 150 mm^2^. A defined pressure was applied by rolling down the test area twice in each lengthwise direction using a 2 kg roller at a rolling speed of 10 mm/s. After a wet-out period of 24 h, the specimens were peeled off at an angle of 180° from the stainless steel plate at a speed of 300 mm/min. The average force during peeling was recorded and used to calculate the peel strength of each sample.

### 2.6. Loop-Tack

Loop-tack tests were conducted on the zwickiLine Z5.0 TN according to the FINAT FTM 9 standard. Test specimens of 25 mm width and 150 mm length (in the machine direction) were cut from the coated PET layer and formed into a loop. The loop was brought into contact with a glass plate on an area of 25 × 25 mm^2^, and was immediately pulled off at a speed of 300 mm/min. The maximum force during the pull-off was recorded and used to calculate the loop-tack of each sample.

## 3. Results and Discussion

### 3.1. Effect of UV-Irradation on Model Adhesive Performance

The effect of crosslinking the base polymers, however initiated, on the adhesive properties is generally known [4,5,6] and was verified in the first step for the model SBS system. Prior to crosslinking, the material exhibits significant tackiness. Upon irradiation, it largely loses its tackiness and develops cohesive strength. The results of the adhesive performance tests of the SBS material are presented in Table 1. UV-initiated crosslinking resulted in an increase in SAFT from 68 °C to 90 °C, while peel strength decreased simultaneously from 22 N/25 mm to 15 N/25 mm. UV irradiation left virtually no tack, decreasing from 24 N/25 mm before irradiation to 1 N/25 mm afterward.

### 3.2. Real-Time Monitoring of Crosslinking Reaction via UV-Rheology

**Figure 1:**
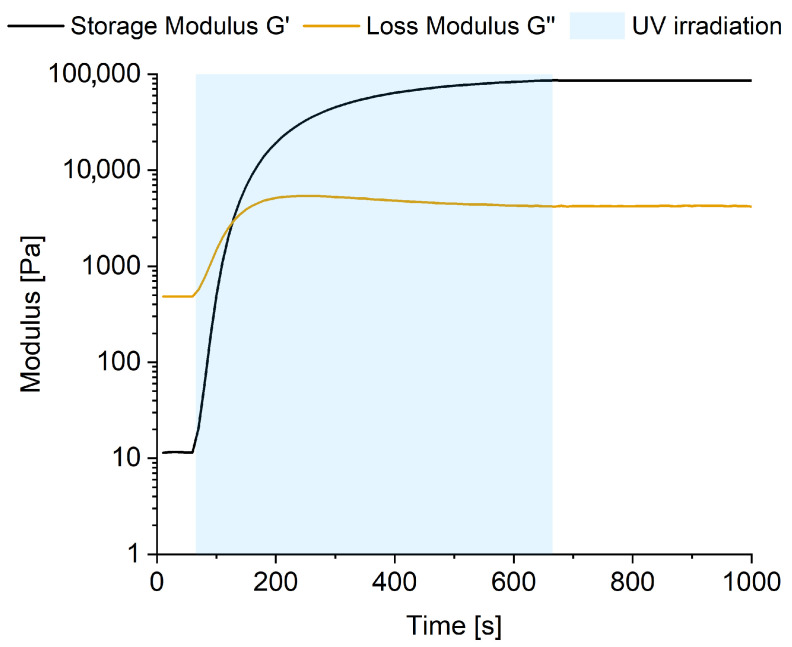
Oscillatory time sweep of SBS model adhesive with simultaneous UV irradiation.

The effects of UV irradiation are not only observable in the final properties of the PSA, but can be monitored online via UV-rheology. Crosslinking was screened by plotting storage modulus G′ and loss modulus G″, measured in oscillatory time sweep measurement on a rheometer with UV accessory. Figure 1 exhibits the impact of UV-irradiation, initiated after 60 s of oscillation, on G′ and G″.

A significant increase in both storage and loss modulus is seen upon UV irradiation. Storage modulus (G′) rises by four orders of magnitude, and loss modulus (G″) by one. Before irradiation, the loss modulus exceeds the storage modulus, indicating the dominance of liquid-like properties. G′ surpasses G″ after 70 s of irradiation, leaving a material with more solid-like viscoelastic properties. After a total of 600 s of UV irradiation, both the storage and loss modulus approach a plateau, exhibiting no further substantial changes over time.

The crosslinking process is presumed to reach completion upon exposure to a UV dose of 5000 mJ/cm^2^, possibly due to the restricted mobility of the now crosslinked polymer chains, or due to the total consumption of the photo initiator used.

### 3.3. Construction of Master Curves Applying Time–Temperature Superposition

To identify potential correlations at frequencies that both align with the adhesive’s potential loads during its lifespan and fall outside the rheometer’s measuring range, the TTS principle was employed to expand the investigable frequency spectrum.

**Figure 2:**
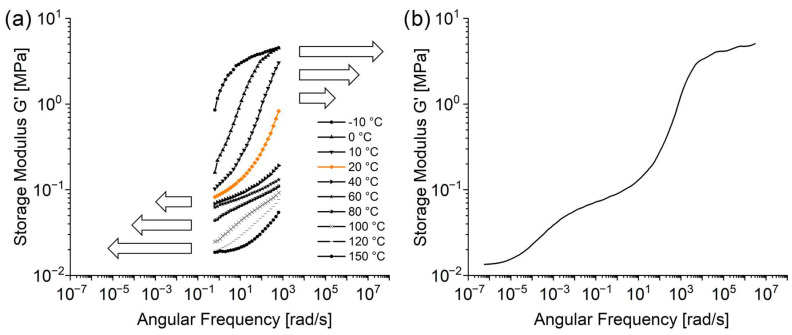
Illustration of the TTS method. (**a**) Original frequency sweep curves of the SBS model adhesive across diverse temperatures. The emphasized graph (orange) at 20 °C serves as the reference for the shift of other results. The ensuing master curve is depicted in (**b**).

To illustrate the TTS method, Figure 2a shows the resulting storage moduli of the frequency sweeps at different temperatures of the irradiated model adhesive. The individual curves were shifted along the *x*-axis around the highlighted graph at the selected reference temperature of 20 °C to generate the single overlapping master curve depicted in Figure 2b.

**Figure 3:**
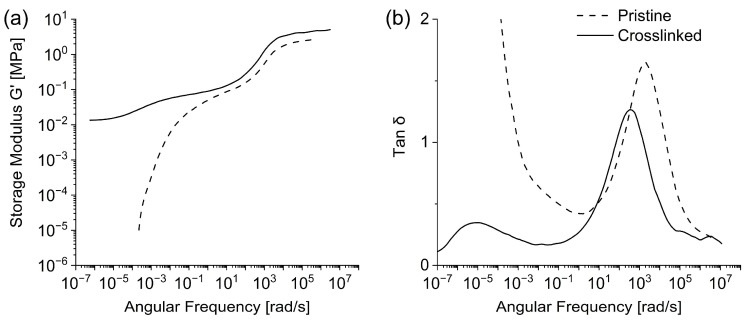
Master curves depicting (**a**) storage modulus G′ and (**b**) loss factor tan δ for the SBS model adhesive pre- and post-UV irradiation.

The curve of tan δ, as well as the results for the pristine material, were treated in the same fashion. The resulting master curves for the SBS model adhesive are displayed in Figure 3, and will be addressed in the subsequent section.

### 3.4. Correlation of Viscoelastic Properties and Adhesive Performance

In practical applications, PSAs endure external loads, evidenced using the evaluated peel, tack, and shear strengths. They react with a blend of reversible (elastic) and irreversible (viscous) deformation. Thus, exploring the viscoelastic properties of PSAs provides valuable insights into predicting their adhesive performance.

The UV-crosslinkable SBS material was selected to discern explicit correlations between viscoelastic properties and adhesive performance, owing to its significant property transformations post-UV irradiation. Examination of the model adhesive’s storage modulus G′ against the angular frequency (refer Figure 3a) reveals that irradiation notably impacts the low frequency range of <1 rad/s. While the non-crosslinked PSA displays a sharply decreasing storage modulus with diminishing angular frequency, the storage modulus of the same irradiated material remains within the same order of magnitude between 0.01 and 0.1 MPa below 1 rad/s. This finding can be related to the change in shear properties triggered by UV irradiation described above.

A PSA’s shear performance is related to its capacity to internally store deformation energy, which is symbolized by its storage modulus G′. Shear load, as applied during the SAFT test, induces slow deformation of the PSA. Therefore, it is the low-frequency region of G′ that is scrutinized to gauge an adhesive’s shear performance. This is further underscored when shear strength is assessed as the SAFT at higher temperatures, which corresponds to lower frequencies according to the TTS principle. Consideration of the model adhesive results thus suggest that storage modulus G′ at low frequencies should be consulted to predict the shear performance of an adhesive. In addition, Figure 3b shows the loss factor tan δ as a function of the angular frequency. Because tan δ results from the ratio of loss modulus and storage modulus, an irradiation-induced increase in the storage modulus G′ significantly reduces the loss factor in the low-frequency range, in contrast to the pristine material.

Moreover, the characteristic peak typically seen at higher frequencies around 10^3^ rad/s also shifts to lower values on both axes following UV irradiation. Given that standard tack tests involve rapid contact and removal of the adhesive from the substrate, bonding and debonding processes occur over a brief timescale. Hence, high-frequency viscoelastic properties are analyzed to understand a PSA’s tack. A higher tan δ indicates the predominance of viscous components over the elastic ones. As such, it is logical that the PSA exhibits fewer viscous properties after UV crosslinking, resulting not only in a drop of maximum tan δ from 1.65 to 1.26, but also in a reduced substrate wettability. Consequently, tack is almost entirely lost, decreasing from 24 N/25 mm to 1 N/25 mm before and after UV irradiation.

The correlation of peel strength and loss modulus G″ postulated elsewhere was also tested for the model adhesive, but could not be established [9,17,22,23].

### 3.5. Commercial UV-Crosslinkable Acrylic PSAs Showing High-Shear, High-Tack, and Intermediate Properties

In order to support the results obtained from the rheological assessment of the model SBS adhesive, commercially available acrylic PSAs showing either high shear, high tack, or intermediate properties were also subjected to identical tests, as previously outlined. The outcomes are shown in Table 2.

#### Storage Modulus G′ and Shear Strength

**Figure 4:**
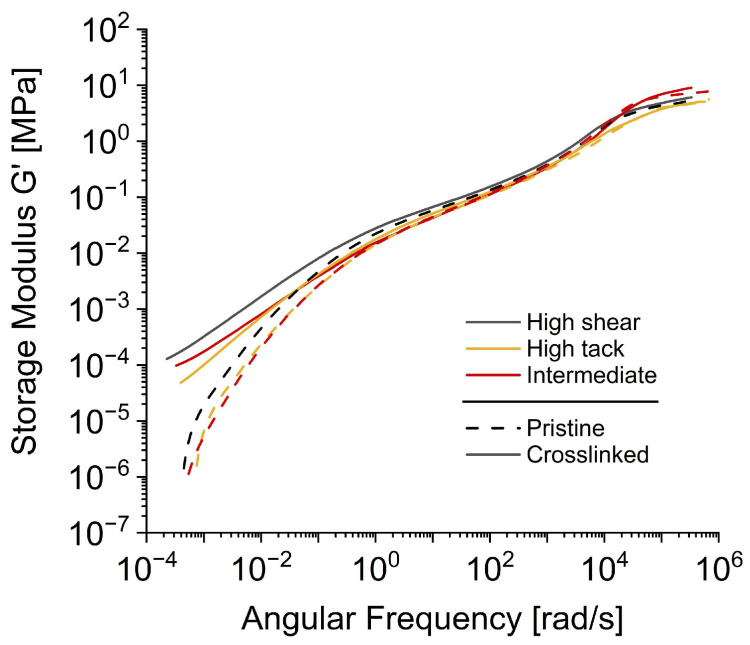
Master curves of the storage moduli for commercial UV crosslinkable PSAs.

Figure 4 depicts the storage moduli of the commercial acrylics, pre- and post-UV irradiation. Mirroring the pattern observed with the model SBS adhesive, commercial PSAs also demonstrate a notable increase in the storage modulus at low frequencies below 1 rad/s following irradiation. However, this change is less significant compared to the model adhesive.

Concurrently, crosslinking enhances the cohesive strength of the commercial products, reflected in increased SAFT. Comparatively, the high-shear adhesive outperforms in terms of cohesive strength, registering a SAFT above 135 °C, while the high tack and intermediate adhesives record SAFT at 108 °C and 119 °C, respectively. This pattern echoes the behavior observed in the model adhesive, and can be traced back to the trajectories of the individual storage modulus curves. Although the intermediate adhesive exhibits the highest overall storage modulus of all compounds at a frequency of 10^5^ rad/s, the SAFT test aligns with a slow stress, and therefore operates at low frequencies. At these low frequencies, less than 1 rad/s, the high-shear adhesive exhibits a marginally superior storage modulus than its high tack and intermediate counterparts. Hence, it is not the highest overall G′, but the G′ at lower frequencies that primarily influences the cohesive strength of PSA materials.

### 3.6. Loss Factor Tan and Tack

**Figure 5:**
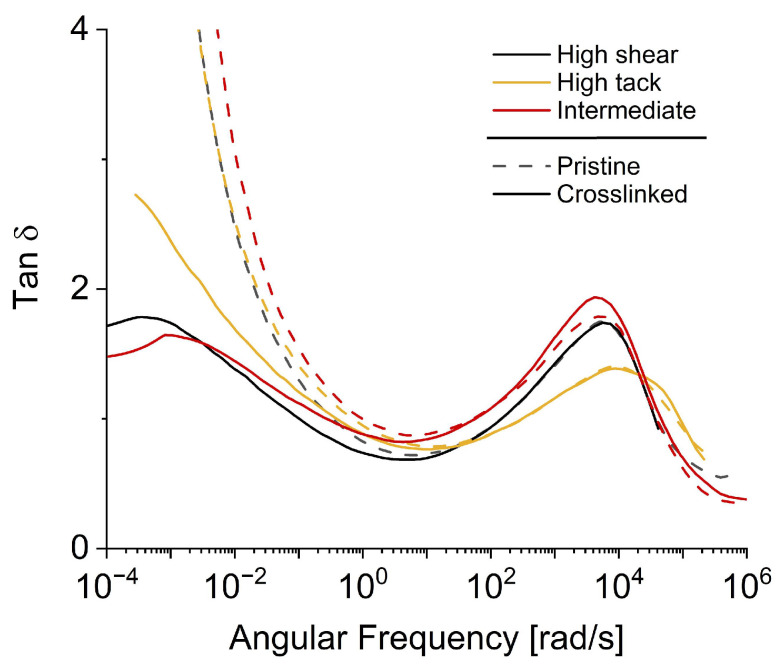
Commercial products’ master curves of tan δ obtained from frequency sweeps.

In the low-frequency domain of tan δ master curves shown in Figure 5, we noted similar effects of irradiation, as previously seen with the model adhesive. The crosslinking and consequent rise in the storage modulus G′ led to a decrease in the loss factor tan δ. However, the shift in the peak observed with the model SBS adhesive was not replicated with the commercial acrylic products. Overall, irradiation had a relatively minor impact on the shape and trajectory of the peak, except for a minor shift noted in the case of the intermediate adhesive, which skewed towards the upper left in contrast to the model adhesive.

A possible explanation for the muted impact of UV irradiation may be a lesser crosslinking capacity or slower crosslinking velocity in commercial acrylates compared to the model SBS adhesive. The structure and concentration of the photoinitiator has an influence on the resulting crosslinking density, as does the interaction of UV emission and absorption by the adhesive. In addition, the possible crosslinking sites are sequentially distributed due to the block structure of the SBS, while the potential crosslinking sites in the polymer backbone of the acrylic products is assumed to be statistically distributed. For instance, if the crosslinking agent’s concentration is lower in commercial products, a less dense polymer network will form upon complete reaction, resulting in less pronounced changes in viscoelastic properties.

When comparing the three products, the results diverge between model SBS and commercial acrylic adhesives. Following irradiation, tack and peel strength only show marginal changes in commercial acrylics compared to the model SBS adhesive. For the SBS adhesive, a high tack coincided with a high loss factor peak maximum. However, the commercial products demonstrated an inverse trend. The high-tack adhesive presented the flattest curve at frequencies > 100 rad/s, exceeded by the loss factors of the intermediate- and high-shear adhesives.

Instead of the peak height, its position on the *x*-axis tended to shift to lower values with decreasing tack, as was observed with the model adhesive. The high-tack adhesive demonstrated the highest tack post-irradiation at 24 N/25 mm, with the tan δ peak around 9000 rad/s. However, both the high-shear and intermediate adhesives displayed lower tack values at 21 and 19 N/25 mm, respectively, with tan δ peaks at lower frequencies, approximately 5500 and 4000 rad/s, respectively.

These varying results indicate that the association between tack and loss factor is not as straightforward as initially inferred from the model SBS adhesive. Predicting an adhesive’s tack performance based on its viscoelastic properties necessitates a more sophisticated model, especially comparing adhesives of different base polymers or different crosslinking properties. This inference is further bolstered by the fact that the correlation between peel strength and loss modulus G″, as reported in the literature, could not be replicated with the tested products [9,17,22,23].

Specifically, peel and tack behaviors necessitate a sophisticated balance of bonding and debonding processes. Numerous attempts have been made to encapsulate this complexity. It is often argued that bonding and debonding happen on separate timescales, and hence distinct frequencies are utilized to evaluate them. Good wettability is usually linked to a low storage modulus at low frequencies. Conversely, an effective energy dissipation, and thus a superior peel strength, is associated with a high loss modulus at high frequencies [24,25,26,27,28].

Yet, none of the models outlined in the literature could furnish a quantitative relationship between viscoelastic properties and adhesive performance for all materials tested when applied to the model SBS and commercial acrylic adhesives. The reasons are multifaceted. Firstly, these models are generally tested and established on well-designed laboratory adhesives with a defined composition. Commercial products, however, typically contain a multitude of components beyond the base polymer, such as fillers, flow aids, tackifiers, and other additives. Secondly, the model adhesive’s chemical base (SBS) and the commercial adhesives (acrylates) differ, as does the UV-triggered crosslinking reaction, which transitions from linear polymer chains to three-dimensional networks. This multitude of influential factors and their interplay make it challenging to encapsulate potential correlations in a single model. Lastly, complex processes occur at the microscopic level during tack and peel tests. Non-linear, discontinuous or surface effects, which cannot be captured by measuring viscoelastic characteristics as bulk properties, play a significant role. These include fibrillation/cavitation [12,15,18,29,30,31], strain hardening [27,31,32], different failure modes such as stick–slip, adhesive, and cohesive failure [8,13,17,18,30,31,32,33], or surface interactions [13,18,30,34].

Our study, employing oscillatory DMA on a model SBS material, uncovered significant correlations between adhesive performance and viscoelastic properties acquired through the TTS principle. UV-triggered crosslinking of the PSA formulation increased cohesive strength, evidenced by a rise in shear adhesion failure temperature (SAFT). Within its viscoelastic parameters, crosslinking led to a higher storage modulus at lower angular frequencies of 1 rad/s and below. Concurrently, peel strength markedly diminished, and tack was nearly entirely eradicated after exposure to UV irradiation. Notably, we observed a slight shift of the tan δ peak towards lower frequencies.

Applying our findings from the model SBS adhesive to commercial UV crosslinkable acrylic PSA products partially validated our observations. However, the effect of UV irradiation on these commercial products was less pronounced. This muted response from the commercial products was anticipated, as the end-use properties of such UV-reactive hotmelt PSA formulations are designed to be finely adjustable through UV irradiation. Nonetheless, the fundamental correlation between shear strength and storage modulus at low frequencies persisted, which is consistent with the relevant literature [22,26].

Our findings on tack, on the other hand, deviated somewhat from previous reports. If employed with the prescribed techniques, rheology could be a potent tool for predicting the end-use performance of PSAs. While our results were less definitive, they suggested a shift of the loss factor tan δ peak toward higher frequencies with better tack. This underscores the necessity of carefully aligning the frequency at which viscoelastic properties are investigated with the velocity and geometry of tack and peel tests.

## 4. Conclusions

Acquiring universally applicable insights about complex adhesive joints by merely examining the adhesive formulation’s bulk properties remains a formidable challenge. While modeling shear stress appears relatively straightforward, predicting tack and peel strength is more intricate, as they emerge from a complex interplay of bonding and debonding processes. Hence, the precise calibration of adhesive and cohesive strength is imperative for achieving optimal properties.

Good and quantitative correlations between viscoelastic properties and bonding performance have been documented with well-designed laboratory adhesives. The TTS principle can facilitate the simulation of specific stress levels by extending the range of evaluable frequencies beyond instrument and method limitations. However, transferring these findings to commercial products with diverse physical structures and chemical compositions and base polymers appears currently unfeasible. Further research is needed to develop not only suitable analytical methods, but also comprehensive models of bonding and debonding processes to render the laborious testing of adhesive performance under real-life conditions obsolete.

## Figures and Tables

**Table 1:** Comparative adhesive performance metrics for the SBS material pre- and post-UV irradiation.

	Pristine	Crosslinked
SAFT [°C]	68	90
Peel [N/25 mm]	22	15
Tack [N/25 mm]	24	1

**Table 2:** Results of the adhesive performance tests of commercial products pre- (pristine) and post-UV irradiation (crosslinked).

	High Shear		High Tack		Intermediate	
	Pristine	Crosslinked	Pristine	Crosslinked	Pristine	Crosslinked
SAFT [°C]	109	>135	53	108	105	119
Peel [N/25 mm]	18	18	29	29	20	19
Tack [N/25 mm]	27	21	33	24	21	19

## Data Availability

Data are contained within the article.
